# Mass spectrometry-based ligand binding assays on adenosine A_1_ and A_2A_ receptors

**DOI:** 10.1007/s11302-015-9477-0

**Published:** 2015-10-19

**Authors:** A. Massink, M. Holzheimer, A. Hölscher, J. Louvel, D. Guo, G. Spijksma, T. Hankemeier, A. P. IJzerman

**Affiliations:** Division of Medicinal Chemistry, LACDR, Leiden University, Leiden, The Netherlands; Division of Analytical Biosciences, LACDR, Leiden University, Leiden, The Netherlands; Leiden Academic Centre for Drug Research, Leiden University, P.O. Box 9502, 2300 RA Leiden, The Netherlands

**Keywords:** MS binding, Mass spectrometry, Radioligand binding, Adenosine receptor, Deuteration

## Abstract

Conventional methods to measure ligand-receptor binding parameters typically require radiolabeled ligands as probes. Despite the robustness of radioligand binding assays, they carry inherent disadvantages in terms of safety precautions, expensive synthesis, special lab requirements, and waste disposal. Mass spectrometry (MS) is a method that can selectively detect ligands without the need of a label. The sensitivity of MS equipment increases progressively, and currently, it is possible to detect low ligand quantities that are usually found in ligand binding assays. We developed a label-free MS ligand binding (MS binding) assay on the adenosine A_1_ and A_2A_ receptors (A_1_AR and A_2A_AR), which are well-characterized members of the class A G protein-coupled receptor (GPCR) family. Radioligand binding assays for both receptors are well established, and ample data is available to compare and evaluate the performance of an MS binding assay. 1,3-Dipropyl-8-cyclopentyl-xanthine (DPCPX) and 4-(2-((7-amino-2-(furan-2-yl)-[1,2,4]triazolo[1,5-*a*]-[1,3,5]triazin-5-yl)amino)ethyl)phenol (ZM-241,385) are high-affinity ligands selective for the A_1_AR and A_2A_AR, respectively. To proof the feasibility of MS binding on the A_1_AR and A_2A_AR, we first developed an MS detection method for unlabeled DPCPX and ZM-241,385. To serve as internal standards, both compounds were also deuterium-labeled. Subsequently, we investigated whether the two unlabeled compounds could substitute for their radiolabeled counterparts as marker ligands in binding experiments, including saturation, displacement, dissociation, and competition association assays. Furthermore, we investigated the accuracy of these assays if the use of internal standards was excluded. The results demonstrate the feasibility of the MS binding assay, even in the absence of a deuterium-labeled internal standard, and provide great promise for the further development of label-free assays based on MS for other GPCRs.

**Electronic supplementary material:**

The online version of this article (doi:10.1007/s11302-015-9477-0) contains supplementary material, which is available to authorized users.

## Introduction

Conventional methods to measure ligand-receptor binding parameters typically require labeled probes such as radiolabeled [1] or fluorescently labeled ligands [2]. Despite the robustness of radioligand binding assays, they carry inherent disadvantages in terms of safety precautions, expensive synthesis, special lab requirements, and waste disposal. Alternatively, the addition of fluorescent moieties holds a substantial risk of affecting the pharmacological properties of a ligand; moreover, in many instances, it is also required to engineer the receptor protein, in particular for fluorescence resonance energy transfer assays [3].

The development of the mass spectrometry (MS) binding assay by the group of Wanner permits to measure binding of an unlabeled ligand to its target [4]. Instead of the radiolabeled ligand in radioligand binding assays, an unlabeled marker ligand is employed in MS binding assays. The amount of marker ligand bound to the target receptor is detected by mass spectrometry. As the mass of the molecule itself is detected, a label is not necessary. However, the marker ligand still has to fulfill the same requirements as radioligands: high affinity and selectivity for the target and low non-specific binding [5]. Therefore, it is practical to choose a ligand for MS binding applications that has already been validated as a good radioligand. This also ensures a straightforward validation of an MS binding assay by comparing it to existing radioligand binding assays.

In this study, we developed an MS binding assay for the adenosine A_1_ (hA_1_AR) and adenosine A_2A_ receptors (hA_2A_AR). The particular robustness and abundance of published results of radioligand binding assays on the hA_1_AR and hA_2A_AR make these receptors good candidates for development of an MS binding assay [6]. The adenosine receptors are members of the class A of G protein-coupled receptors (GPCRs). Both receptors are important in physiology. The hA_1_AR has been related to sleep regulation, epilepsy, and asthma. The hA_2A_AR is implicated in neurodegeneration, inflammatory diseases, and cancer pathogenesis. Both receptors are involved in cardiovascular physiology [6, 7]. As marker ligands for the MS binding assay, we chose 1,3-dipropyl-8-cyclopentyl-xanthine (DPCPX) for the hA_1_AR and 4-(2-((7-amino-2-(furan-2-yl)-[1,2,4]triazolo[1,5-*a*]-[1,3,5]triazin-5-yl)amino)ethyl)phenol (ZM-241,385) for the hA_2A_AR. These ligands are well-established radioligands for their respective targets and hence a logical choice to serve as marker ligands in MS binding assays [8, 9].

The development of liquid chromatography-MS (LC-MS) detection methods for non-labeled DPCPX and ZM-241,385 as marker ligands involved the following steps. Firstly, deuterated isotopologues of the marker ligands were synthesized to serve as internal standards for increased accuracy of the MS detection. In MS detection methods, it is common to add a fixed amount of an internal standard to each sample to compensate for ion suppression, sample evaporation, and instrumental drift [10]. Technically, the use of deuterium-labeled internal standards makes the MS binding assay a labeled assay, even if the marker ligand that binds to the target is unlabeled itself. Therefore, we also investigated whether the results of the MS binding assays were accurate in the absence of an internal standard. Secondly, a fast LC method was developed to separate the marker ligands from cell membrane contents in the sample. The duration of the LC separation is the limiting step for the throughput of the method so this is preferably fast, i.e., within 1 min. Thirdly, for MS detection, a triple quadrupole MS (TQMS) was employed, which has the required sensitivity to measure typical bound ligand quantities of ligand binding assays, in the pM range. In a TQMS, the parent ions with the mass of the molecule of interest are filtered by the first quadrupole, which are then fragmented in the second quadrupole. The fragmentation results in daughter ions that are analyzed by the third quadrupole. This setup ensures a high selectivity and sensitivity for the detection of a molecule of interest [11].

After establishing the LC-MS methods for detection of the marker ligands, the MS binding assays were performed with and without deuterium-labeled internal standard, and analogous to radioligand binding assays. Saturation, association, and dissociation assays were performed to determine the affinity and kinetic rates of the marker ligands DPCPX for the hA_1_AR and ZM-241,385 for the hA_2A_AR. Then displacement and competition association assays were performed to determine the affinity and kinetic rates of ligands competing with the marker ligands. The ensuing results were compared to and validated with reference radioligand binding data.

## Materials and methods

### Materials

Adenosine deaminase (ADA) was purchased from Boehringer Mannheim (Mannheim, Germany). DPCPX, 5′-*N*-ethylcarboxamidoadenosine (NECA), and bovine serum albumin (BSA) were purchased from Sigma (St. Louis, MO, USA). ZM-241,385 was purchased from Ascent Scientific (Bristol, UK). *N*^6^-Cyclopentyladenosine (CPA) was purchased from Abcam Biochemicals (Cambridge, UK). 6-(2,2-Diphenylethylamino)-9-((2*R*,3*R*,4*S*,5*S*)-5-(ethylcarbamoyl)-3,4-dihydroxytetrahydrofuran-2-yl)-*N*-(2-(3-(1-(pyridin-2-yl)piperidin-4-yl)ureido)ethyl)-9*H*-purine-2-carboxamide (UK-432,097) was obtained as a gift through Pfizer’s Compound Transfer Program. 3-(3-Hydroxypropyl)-7-methyl-1-propargyl-8-(*m*-methoxystryryl)xanthine (MSX-2) [12] was a gift from Prof. C. E. Müller (Bonn University, Germany). 8-Cyclopentyltheophylline (8-CPT) was purchased from Research Biochemicals Inc. (Natick, MA, USA). 8-Cyclopentyl-3-(3-((4-(fluorosulfonylbenzoyl)oxy)propyl)-1-propylxanthine (FSCPX) [13] and *N*^5^-(2-(4-(2,4-difluorophenyl)piperazin-1-yl)ethyl)-2-(furan-2-yl)-[1,2,4]triazolo[1,5-*a*][1,3,5]triazine-5,7-diamine (LUF6632) [14] were synthesized in-house. Bicinchoninic acid (BCA) and BCA protein assay reagent were obtained from Pierce Chemical Company (Rockford, IL, USA). CHO cells stably expressing the hA_1_AR (CHO-hA_1_AR) were a gift from Prof. S. Hill (University of Nottingham, UK). HEK293 cells stably expressing the hA_2A_AR (HEK293-hA_2A_AR) were a gift from Dr. J. Wang (Biogen/IDEC, Cambridge, MA, USA). All other chemicals were of analytical grade and obtained from standard commercial sources.

### General synthesis procedures

Demineralised water is simply referred to as H_2_O, as was used in all cases unless stated otherwise. ^1^H and ^13^C NMR spectra were recorded on a Bruker AV 400 liquid spectrometer (^1^H NMR, 400 MHz; ^13^C NMR, 100 MHz) at ambient temperature. Chemical shifts are reported in parts per million (ppm), are designated by δ, and are downfield to the internal standard tetramethylsilane (TMS) in CDCl_3_. Coupling constants are reported in Hertz and are designated as *J*. Analytical purity of the final compounds was determined by high pressure liquid chromatography (HPLC) with a Phenomenex Gemini 3-μm C18 110A column (50 × 4.6 mm, 3 μm), measuring UV absorbance at 254 nm. Sample preparation and HPLC method were—unless stated otherwise—as follows: 0.3–0.8 mg of compound was dissolved in 1 ml of a 1:1:1 mixture of CH_3_CN/H_2_O/tBuOH and eluted from the column within 15 min, with a three-component system of H_2_O/CH_3_CN/1 % TFA in H_2_O, decreasing polarity of the solvent mixture in time from 80:10:10 to 0:90:10. All compounds showed a single peak at the designated retention time and were at least 95 % pure. The synthesized compounds were identified by LC-MS analysis using a Thermo Finnigan Surveyor-LCQ Advantage Max LC-MS system and a Gemini C18 Phenomenex column (50 × 4.6 mm, 3 μm). The sample preparation was the same as for HPLC analysis. The elution method was set up as follows: 1–4 min isocratic system of H_2_O/CH_3_CN/1 % TFA in H_2_O, 80:10:10, from the fourth minute, a gradient was applied from 80:10:10 to 0:90:10 within 9 min, followed by 1 min of equilibration at 0:90:10 and 1 min at 80:10:10. Thin-layer chromatography (TLC) was routinely performed to monitor the progress of reactions, using aluminum-coated Merck silica gel F254 plates. Purification by column chromatography was achieved by use of Grace Davison Davisil silica column material (LC60A 30–200 μm). Solutions were concentrated using a Heidolph laborota W8 2000 efficient rotary evaporation apparatus and by a high vacuum on a Binder APT line Vacuum Drying Oven.

### Preparation of 8-cyclopentyl-1,3-bis(propyl-2,3-d_2_)-3,9-dihydro-1*H*-purine-2,6-dione 2 ([^2^H_4_]DPCPX)

Synthesis steps to arrive to compound 1 (SI Scheme 3) were performed as previously described [15–17]. 1,3-Diallyl-8-cyclopentyl-3,9-dihydro-1*H*-purine-2,6-dione 1 (1 mmol, 300 mg) and NaBD_4_ (4 mmol, 167 mg) were placed in a flask. The flask was flame-dried under vacuum to remove traces of water and then purged with N_2_ gas. Dry THF (10 ml) was added. RhCl(PPh_3_)_3_ was placed in another flame-dried flask under N_2_-atmosphere and suspended in dry THF (1 ml). The flask containing 1 was heated to 60 °C, and the reaction was started upon addition of the catalyst suspension, followed by D_2_O (2 mmol, 0.04 ml). The mixture was stirred at 60 °C for 19 h. The reaction mixture was then poured into EtOAc and washed with brine (3×). The organic layer was dried over MgSO_4_ and concentrated. The crude product was purified by column chromatography (PET/EtOAc 5/1 → 4/1 → 3/2). The product 2 was obtained as white solid (46 %, 0.46 mmol, 141 mg). ^1^H NMR (400 MHz, CDCl_3_): δ 11.92 (br s, 1H), 4.11–4.06 (m, 2H), 4.03–3.97 (m, 2H), 3.30–3.21 (m, 1H), 2.18–2.11 (m, 2H), 1.98–1.68 (m, 8H), 0.99–0.94 (m, 4H) ppm. MS: [M + H]^+^ calculated 309.22, found 309.20. HPLC purity 97 % (*t*_R_ 9.587 min, mobile phase 15–65 % MeCN/H_2_O + TFA).

### Preparation of 4-(2-((7-amino-2-(furan-2-yl)-1,3a-dihydro-[1,2,4]triazolo[1,5-a][1,3,5]triazin-5-yl)amino)ethyl)phen-2,3,5,6-d_4_-ol 5 ([^2^H_4_]ZM-241,385)

Synthesis steps to arrive to compounds 3 and 4 (SI Scheme 4) were performed as previously described [18–20]. [^2^H_4_]Tyramine 4 (0.37 mmol, 53 mg) was suspended in 4 ml MeCN, and Et_3_N (0.14 ml) and 2-(furan-2-yl)-5-(methylsulfonyl)-1,3a-dihydro-[1,2,4]triazolo[1,5-a][1,3,5]triazin-7-amine 3 (0.34 mmol, 95 mg) were added. The mixture was stirred for 3 h at 70 °C under microwave irradiation. The solvent was evaporated, and the crude material was adsorbed onto silica and purified by column chromatography (EtOAc/MeOH 99/1) and subsequent PTLC (EtOAc/MeOH 99.5/0.5) to give the product 5 as an off-white solid (45 %, 0.15 mmol, 52 mg). ^1^H NMR (400 MHz, DMSO-*d*6): δ 9.17 (s, 1H), 8.13 (br s, 2H), 7.86 (s, 1H), 7.53–7.52 and 7.50–7.42 (m, 1H, rotamers), 7.05 (d, *J* = 3.2, 1H), 6.67 (m, 1H), 3.43–3.40 (m, 2H), 2.74–2.71 (m, 2H) ppm. MS: [M + H]^+^ calculated 342.16, found 342.7. HPLC purity 95 % (*t*_R_ 6.408 min, mobile phase 10–90 % MeCN/H_2_O + TFA).

### Cell culture

CHO-hA_1_AR cells were grown in Ham’s F12 medium containing 10 % normal adult bovine serum, 100 μg/ml streptomycin, 100 IU/ml penicillin, and 400 μg/ml G418, at 37 °C in 5 % CO_2_. HEK293-hA_2A_AR cells were grown in Dulbecco’s modified Eagle’s medium (DMEM) supplemented with 10 % newborn calf serum, 50 μg/ml streptomycin, 50 IU/ml penicillin, and 200 μg/ml G418, at 37 °C and 7 % CO_2_. Cells were subcultured twice a week on 10-cm ø plates at a ratio of 1:20 for CHO hA_1_R cells and 1:8 for HEK293 hA_2A_AR cells.

### Membrane preparation

CHO-hA_1_AR and HEK293-hA_2A_AR cells were grown as described above. Membranes were prepared as follows. Cells were detached from plates grown to confluency by scraping them into 5 ml PBS, collected and centrifuged at 700*g* (3000 rpm) for 5 min. Pellets derived from 20 plates (10 cm ø) were pooled and resuspended in 16 ml of ice-cold assay buffer (50 mM Tris-HCl, 5 mM MgCl_2_, pH 7.4). An Ultra-Turrax was used to homogenize the cell suspension. Membranes and the cytosolic fraction were separated by centrifugation at 100,000*g* (31,000 rpm) in a Beckman Optima LE-80K ultracentrifuge at 4 °C for 20 min. The pellet was resuspended in 8 ml of Tris buffer and the homogenization and centrifugation step was repeated. Assay buffer (4 ml) was used to resuspend the pellet, and adenosine deaminase (ADA) was added (0.8 IU/ml) to break down endogenous adenosine. Membranes were stored in 250-μl aliquots at −80 °C. Membrane protein concentrations were measured using the BCA (bicinchoninic acid) method [21].

### Radioligand binding assays

The reference radioligand binding data were published before by our lab or were acquired as described before [22, 23].

### Membrane harvesting procedure MS binding assays

One hundred-microliter membrane aliquots containing 5 μg (CHO-hA_1_AR) or 22 μg (HEK293-hA_2A_AR) of protein in assay buffer were harvested by rapid vacuum filtration through 1-μm glass fiber AcroPrep Advance 96 filter plates (Pall Corporation, Ann Arbor, MI, USA) using an extraction plate manifold (Waters, Milford, MA, USA) and a 12-channel electronic pipette (Gilson, Middleton, WI, USA). Filters were subsequently washed three times with ice-cold assay buffer and dried for 1 h at 55 °C. It was essential that the filter plates were completely dry before continuing with ligand elution as described below in “Sample elution.”

### MS binding saturation assays

Membrane aliquots containing 5 μg (CHO-hA_1_AR) or 22 μg (HEK293-hA_2A_AR) of protein were incubated in a total volume of 100 μl of assay buffer at 25 °C for 1 h (hA_1_AR) or at 4 °C for 3 h (hA_2A_AR). Total binding was determined at increasing concentrations of marker ligand DPCPX (0.08–40 nM on hA_1_AR) or marker ligand ZM-241,385 (0.05–15 nM on hA_2A_AR). Dilutions were prepared with a HP D300 Digital Dispenser (Tecan Group, Männerdorf, Swiss) from DMSO stocks. Non-specific binding in presence of 100 μM CPA (hA_1_AR) or 100 μM NECA (hA_2A_AR) was determined at three concentrations of marker ligand and analyzed by linear regression. Incubations were terminated and samples were harvested as described under “Membrane harvesting procedure MS binding assays.”

### MS binding displacement assays

Ligand displacement experiments were performed using nine concentrations of competing ligand. For the hA_1_AR, the competing ligands used were CPA, 8-CPT, ZM-241,385, and NECA, while for the hA_2A_AR, they were UK-432,097, MSX-2, DPCPX, and NECA. As marker ligand DPCPX was used for the hA_1_AR at a concentration of 6 nM, and ZM-241,385 for the hA_2A_AR at a concentration of 3 nM. Non-specific binding was determined in the presence of 100 μM CPA for the hA_1_AR and 100 μM NECA for the hA_2A_AR. Incubations were terminated as described under “Membrane harvesting procedure MS binding assays.”

### MS binding association assays

Membrane aliquots containing 5 μg/100 μl (CHO-hA_1_AR) or 22 μg/100 μl (HEK293-hA_2A_AR) of protein were incubated in a total volume of 2400 μl of assay buffer at 25 °C with 6 nM DPCPX for hA_1_AR or at 4 °C with 3 nM ZM-241,385 for hA_2A_AR. At each time point, 100 μl from the reaction mix was harvested as described under “Membrane harvesting procedure MS binding assays” to determine the amount of marker ligand bound to the receptor. Non-specific binding was determined as described under “MS binding displacement assays.”

### MS binding dissociation assays

Membrane aliquots containing 5 μg/100 μl (CHO-hA_1_AR) or 22 μg/100 μl (HEK293-hA_2A_AR) of protein were incubated in a total volume of 2400 μl of assay buffer at 25 °C with 6 nM DPCPX (hA_1_AR) or at 4 °C with 3 nM ZM-241,385 (hA_2A_AR). The reaction mixes were allowed to reach equilibrium for 1 h before starting the dissociation by adding 100 μM CPA (hA_1_AR) or NECA (hA_2A_AR). At each time point, 100 μl from the reaction mix was harvested as described under “Membrane harvesting procedure MS binding assays” to determine the amount of marker ligand still bound to the receptor. Non-specific binding was determined as described under “MS binding displacement assays.”

### MS binding competition association assays

Membrane aliquots containing 5 μg/100 μl (CHO-hA_1_AR) or 22 μg/100 μl (HEK293-hA_2A_AR) of protein were incubated in a total volume of 2400 μl of assay buffer at 25 °C with 6 nM DPCPX in the absence or presence of 250 nM 8-CPT or 250 nM FSCPX (hA_1_AR) or at 4 °C with 3 nM ZM-241,385 in the absence or presence of 90 nM MSX-2 or 15 nM LUF6632 (hA_2A_AR). At each time point, 100 μl from the reaction mix was harvested as described under “Membrane harvesting procedure MS binding assays” to determine the amount of marker ligand bound to the receptor. Non-specific binding was determined as described under “MS binding displacement assays.”

### Sample elution

The ligand was eluted from the ligand-receptor complex on the dried filter plates over which MS binding samples were harvested. One hundred microliters of eluent (50 % methanol, 50 % ammonium formate buffer [final concentration 5 mM] at pH 7, spiked with 2 nM [^2^H]DPCPX or [^2^H]ZM-241,385 as internal standard, all HPLC grade) was applied to the filter plates which were then centrifuged for 1 min at 800*g* (2000 rpm) in a 5810 plate centrifuge (Eppendorf, Hamburg, Germany), while filter eluates were collected in 1.1-ml polystyrene deep 96-well plates (BrandTech Scientific, Essex, CT, USA). This procedure was performed twice resulting in a total of 200 μl eluate for each sample. For standard curve samples, the same procedure was followed but for the presence of increasing concentrations (1–100 pM) of DPCPX or ZM-241,385 in the eluent. After elution, 96-deep-well plates were sealed with rapid easy pierce film (Nacalai, San Diego, CA, USA) and stored at −20 °C before LC-MS-MS quantification.

### LC-MS-MS quantification

All solvents used were of LC-MS grade or better. The LC-ESI-MS-MS setup consisted of a Nexera X2 UHPLC (Shimadzu, Kyoto, Japan; degassing unit: 20A3R, autosampler: 30AC, column oven: 30AD) and a LCM-8050 triple quadrupole mass spectrometer (Shimadzu, Kyoto, Japan) with an electrospray ionization source (ESI) in positive mode. Chromatographic separation was performed on an Acquity UPLC BEH C18 column (1 × 50 mm, 1.7 μm; Waters, Milford, MA, USA) with a VanGuard precolumn of the same type (2.1 × 5 mm). The column oven was set at 40 °C. Mobile phases consisted of acetonitrile, methanol, and ammonium formate buffer (final concentration 5 mM) at pH 7, all of LC-MS grade or better, in respective volume fractions of 5:5:90 (solvent A) and 45:45:10 (solvent B). An isocratic mobile phase flow of 0.2 ml/min was applied consisting of solvents A:B (10:90 for the DPCPX and 35:65 for the ZM-241,385 quantification methods), which resulted in column pressures of 400 and 500 bar, respectively. The sample eluate injection volume was 20 μl and run time was 1 min. Source and fragmentation parameters were acquired by the Shimadzu optimization for method function (Table 1). For each ligand, the parent and four daughter ions were detected by multiple reaction monitoring (MRM) in positive mode. Additional MS settings were as follows: ESI interface temperature 300 °C; DL temperature 250 °C; heat block temperature 400 °C; ion spray voltage 4 kV; heating and drying gas flows 10 l/min; nebulizing gas flow 3 l/min.

**Table 1:** Mass of detected parent and daughter ions. Parent ions were fragmented to daughter ions with different optimal collision energies for each daughter ion

	Parent ion	Daughter ion	Collision energy (V)
DPCPX	305.00	178.15	35
		204.10	36
		221.15	27
		263.10	23
[^2^H]DPCPX	309.00	178.10	36
		204.05	35
		221.05	29
		265.00	23
ZM-241,385	338.10	77.05	55
		121.05	29
		176.10	30
		218.10	24
[^2^H]ZM-241,385	342.10	80.05	65
		125.10	30
		176.05	32
		218.05	26

### Data analysis

Shimadzu LabSolutions software (Shimadzu, Kyoto, Japan) was used to analyze resulting chromatogram peaks. The peak area of the total ion count (TIC) of the daughter ions was calculated at the expected retention times, resulting in marker and internal standard peak area. To compensate for eluent evaporation and signal suppression by matrix effects from the membrane sample, marker peak area was divided by internal standard peak area (M/IS). Lower limit of quantitation (LLOQ) values of each marker ligand were defined as the lowest concentration in membrane matrix where signal to noise ratio was higher than 5, the standard deviation within and between runs in hexaplicate was lower than 20 % (and for all higher concentrations lower than 15 %), and calculation of concentration by a function derived from 1/*x*^2^ linear regression deviated from nominal values less than 20 %. M/IS values were converted to concentration of marker ligand in pM using the function established by 1/*x*^2^ linear regression on the 10–100-pM standard curve results. The resulting MS binding data was then analyzed with GraphPad Prism 5.0 (GraphPad Software Inc., San Diego, CA, USA). Marker ligand displacement curves were fitted to one- and two-state site binding models. *k*_on_ and *k*_off_ values of the marker ligands DPCPX and ZM-241,385 were derived by fitting one-phase association and dissociation models. Association and dissociation rates for the competing ligands were calculated by fitting the data to the competition association model using “kinetics of competitive binding” [24]. Log-transformed *K*_i_, *K*_D_, *k*_on_, and *k*_off_ values from MS binding and radioligand binding assays were plotted, and a linear regression analysis was applied. A similar correlation plot was prepared with values from MS binding assays based on solely marker peak area values instead of M/IS.

## Results

### Synthesis of [^2^H_4_]DPCPX 2

**Scheme 1:**
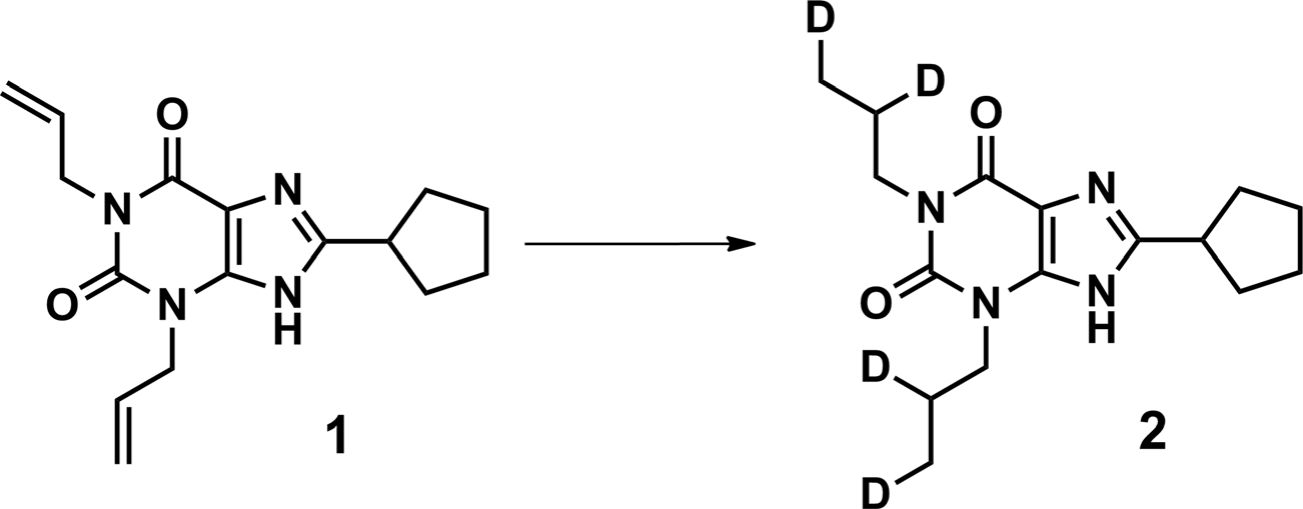
Synthesis of [^2^H_4_]DPCPX (2). Reagents and conditions: Rh(PPh_3_)_3_Cl, NaBD_4_, D_2_O, dry THF, 60 °C, 3.5 h

[^2^H_4_]DPCPX 2 was prepared according to the synthetic route shown in Scheme 1 and SI Scheme 3 and was adopted from previously described syntheses of non-deuterated DPCPX [15–17]. After the synthesis steps to arrive at compound 1 (SI Scheme 3), the allylic double bonds of the DPCPX precursor 1 were reductively deuterated in the presence of Wilkinson’s catalyst with NaBD_4_ as a deuterium source generating deuterium gas in situ upon addition of D_2_O [25]. The mass spectrum showed a mass range for the (M + H^+^) species from 305.20 ([^2^H_0_] isotopologue) to 313.27 ([^2^H_8_] isotopologue) in a Gaussian distribution with the desired [^2^H_4_]DPCPX 2 as most abundant isotopologue generating the main mass peak at 309.33.

### Synthesis of [^2^H_4_]ZM-241,385 5

**Scheme 2:**
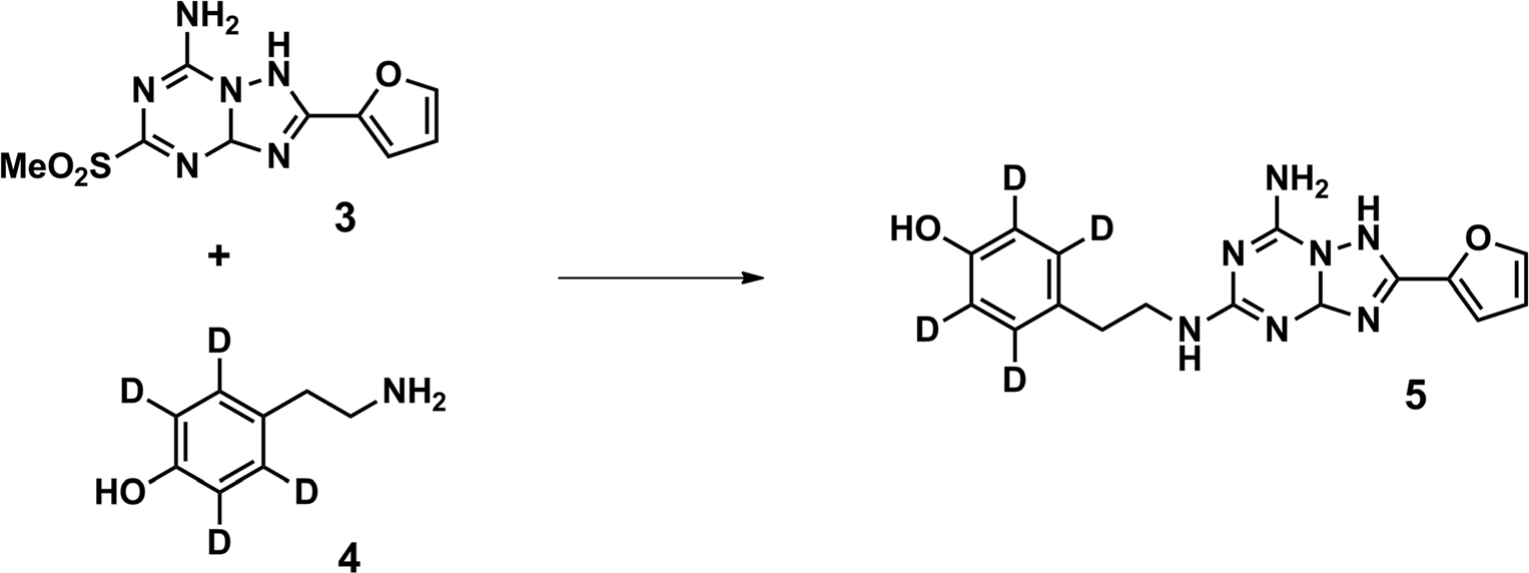
Synthesis of [^2^H_4_]ZM-241,385 (5). Reagents and conditions: Et_3_N, MeCN, MW, 70 °C, 3 h

[^2^H_4_]ZM-241,385 5 was prepared according to the synthetic route shown in Scheme 2 and SI Scheme 4 and was adopted from previously described syntheses of non-deuterated ZM-241,385 [18–20]. After the synthesis steps to arrive at compounds 3 and 4 (SI Scheme 4), reaction of the [^2^H_4_]tyramine 4 with methylsulfone compound 3 yielded the final product [^2^H_4_]ZM-241,385 5 [18]. MS analysis showed a mass of 342.7 (M + H^+^) and confirmed the incorporation of four deuterium atoms in the final product.

### LC-MS results of DPCPX, [^2^H]DPCPX, ZM-241,385, and [^2^H]ZM-241,385

**Fig. 1:**
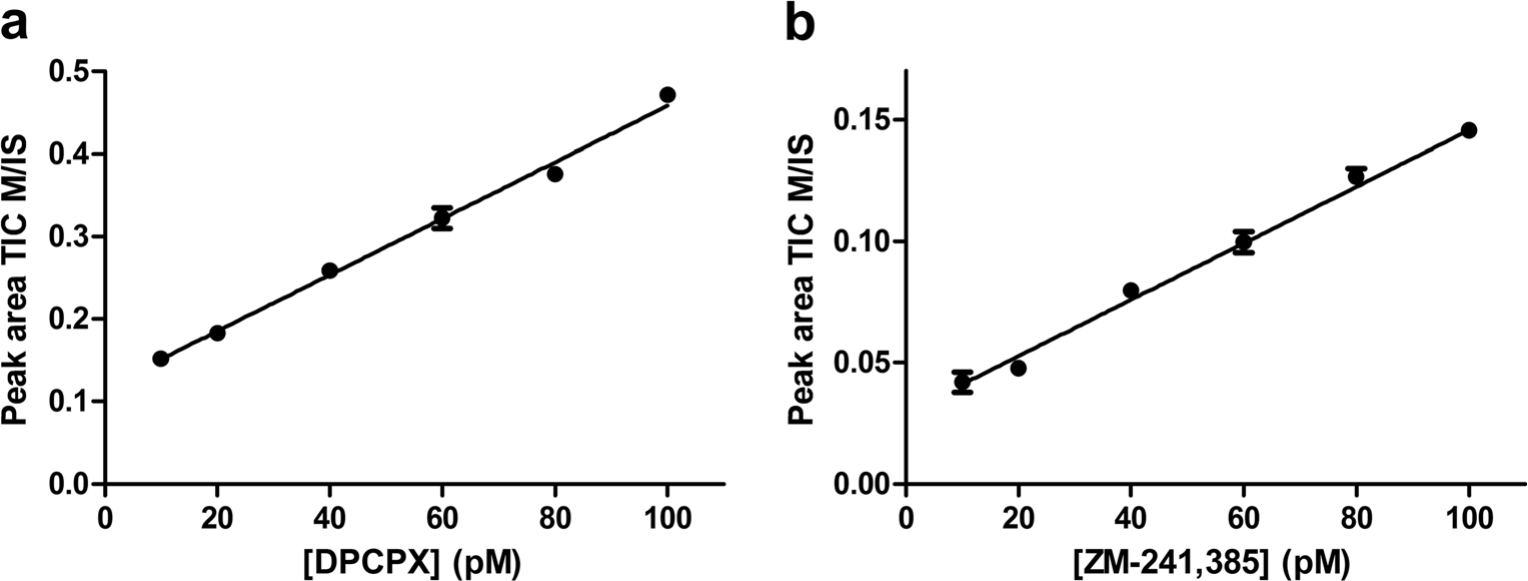
Standard curve of increasing concentrations of marker ligands **a** DPCPX with 2 nM [^2^H]DPCPX and **b** ZM-241,385 with 2 nM [^2^H]ZM-241,385 in matrix membrane samples. On the *x*-axis is plotted the concentration of marker ligand. On the *y*-axis is plotted the marker area TIC divided by IS area TIC (M/IS). Data shown is the average of M/IS values ± SEM from four runs in hexaplicate

Standard curves were made for the quantitation of DPCPX and ZM-241,385 concentrations in biological membrane matrix by LC-MS (Fig. 1). Membrane samples without addition of ligand were filtered, and the applied eluent contained increasing concentrations of DPCPX and ZM-241,385, in addition to 2 nM of their deuterated counterparts. This method ensured standard curves in presence of the same biological matrix as for the quantitated MS binding samples. The LLOQ values derived from the standard curves were 20 pM for DPCPX and 40 pM for ZM-241,385 and were below non-specific binding concentrations found in MS binding assays for DPCPX (31 pM) and ZM-241,385 (42 pM). The linear regression equations to calculate marker ligand concentrations from the M/IS values derived from the standard curves were *y* = 0.00341*x* + 0.117 with *R*^2^ = 0.988 for DPCPX and *y* = 0.00130*x* + 0.0225 with *R*^2^ = 0.987 for ZM-241,385.

### Binding assays

**Fig. 2:**
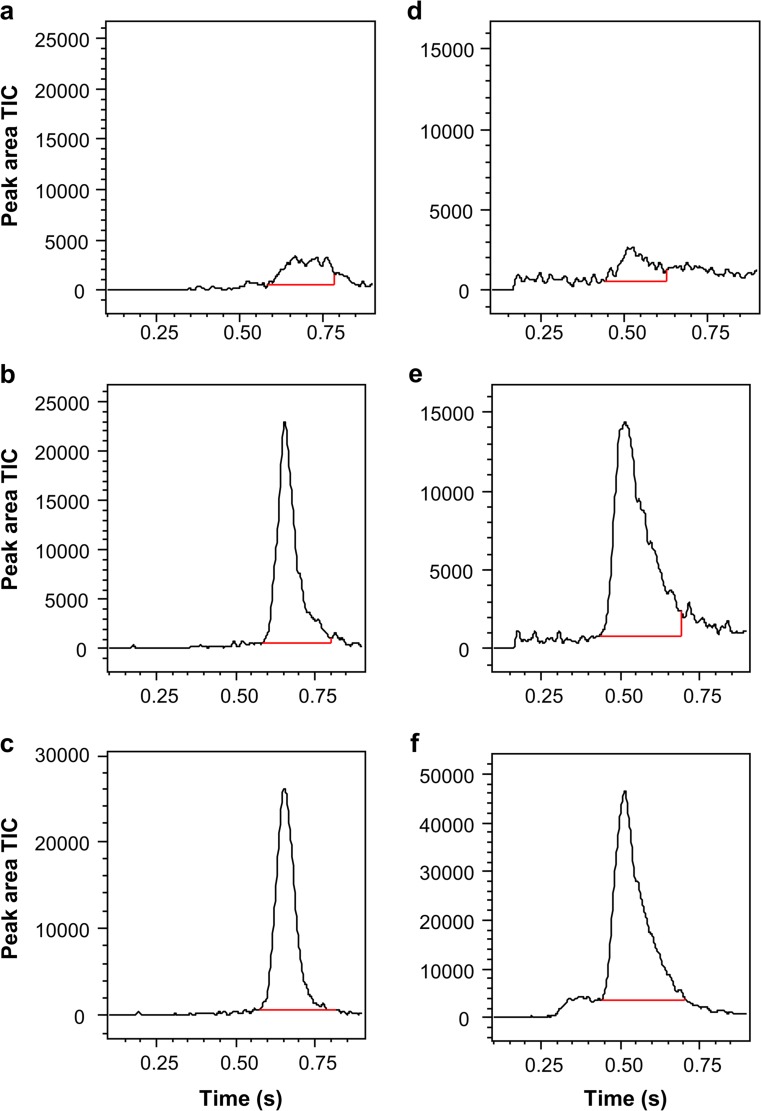
Typical chromatograms of **a** non-specific binding of DPCPX (31 pM), **b** total binding of DPCPX (242 pM), and **c** [^2^H]DPCPX (2 nM) in eluate containing hA_1_AR membrane matrix, and of **d** non-specific binding of ZM-241,385 (42 pM), **e** total binding of ZM-241,385 (289 pM), and **f** [^2^H]ZM-241,385 (2 nM) in eluate containing hA_2A_AR membrane matrix. The *red lines* delineate the area of peak integration

**Fig. 3:**
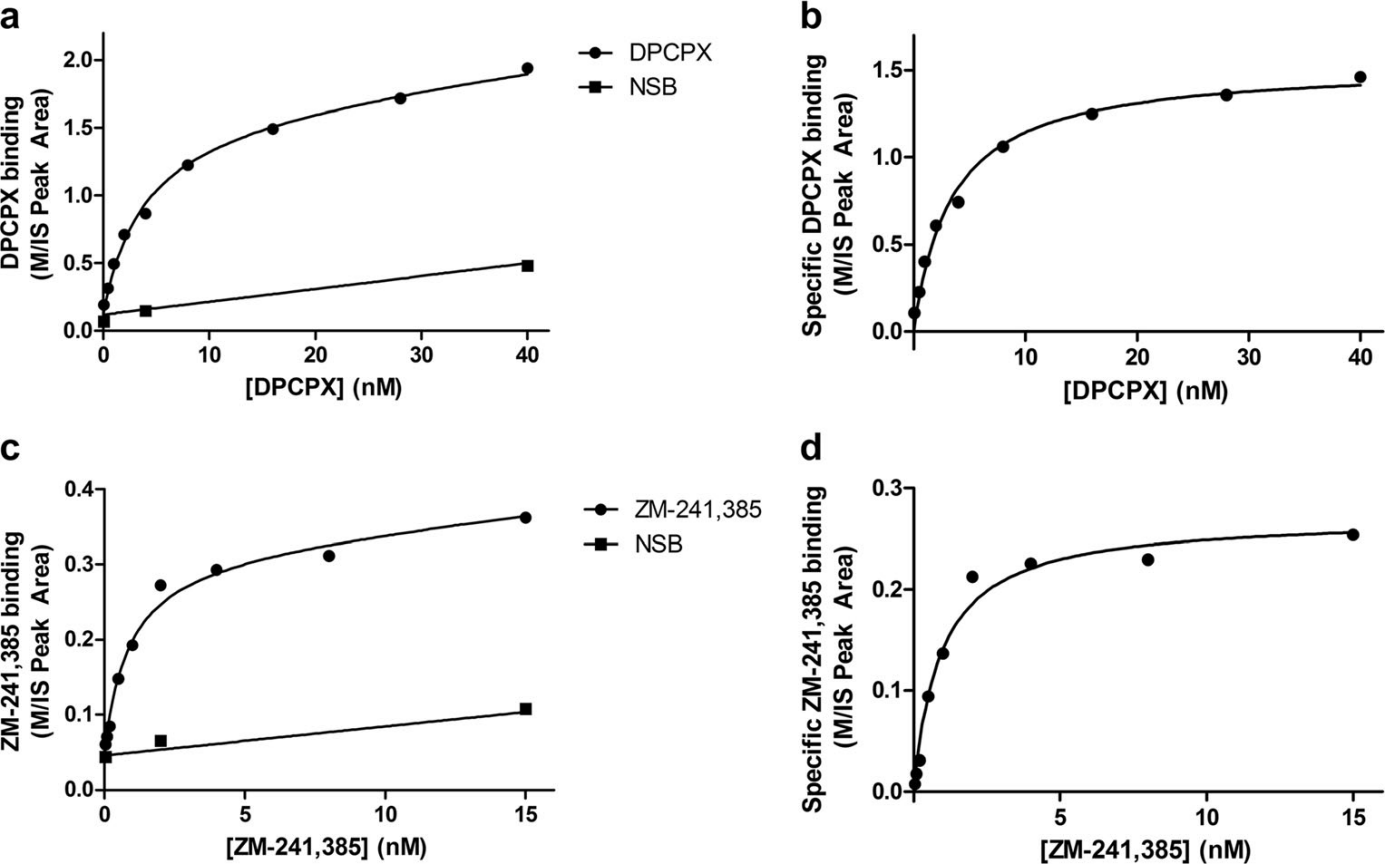
Saturation of DPCPX binding to hA_1_AR (**a**, **b**) and ZM-241,385 binding to hA_2A_AR (**c**, **d**). Increasing concentrations of marker ligands were incubated with the respective membranes. Data shown without (**a**, **c**) and with (**b**, **d**) non-specific binding values subtracted. Graphs show mean values of one representative MS binding saturation experiment performed in duplicate

**Fig. 4:**
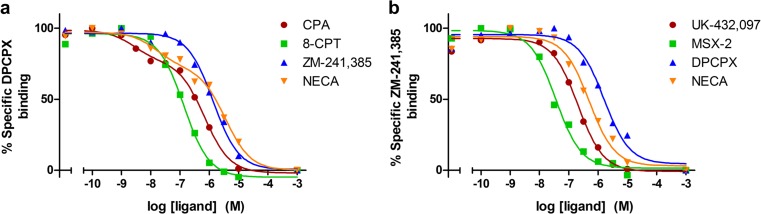
Displacement of DPCPX binding to hA_1_AR by CPA, 8-CPT, ZM-241,385, or NECA (**a**) and of ZM-241,385 binding to hA_2A_AR by UK-432,097, MSX-2, DPCPX, or NECA (**b**). Non-specific binding is plotted at −3 on the *x*-axis. Graphs show mean values of one representative MS binding displacement experiment performed in duplicate

**Fig. 5:**
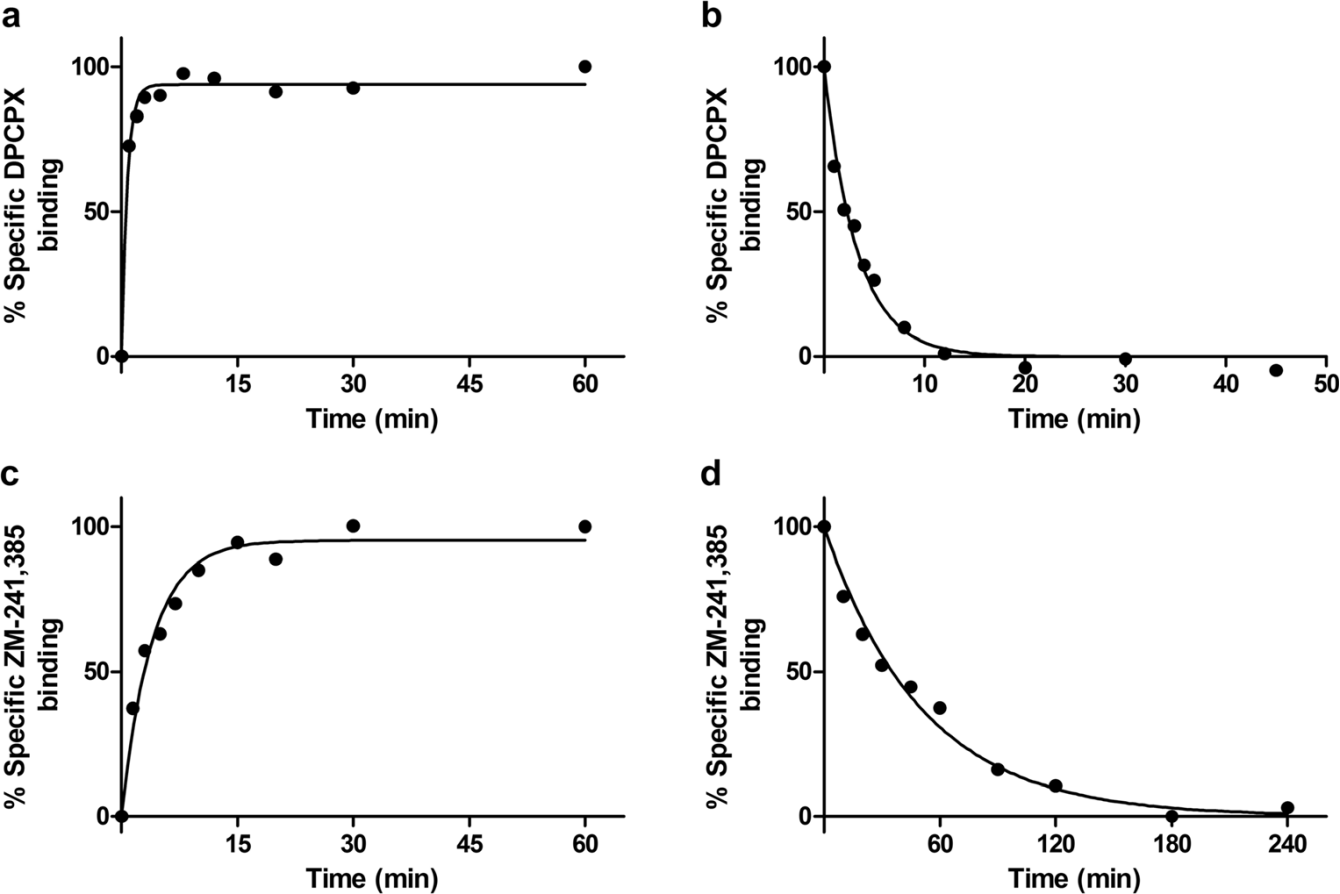
Association (**a**, **c**) and dissociation (**b**, **d**) of DPCPX on hA_1_AR (**a**, **b**) and ZM-241,385 on hA_2A_AR (**c**, **d**). Graphs show mean values of one representative MS binding association or dissociation experiment performed in duplicate

**Fig. 6:**
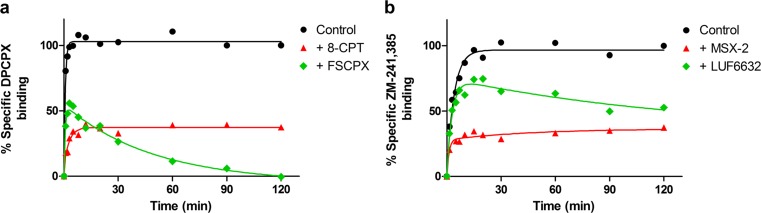
Competition association of DPCPX on hA_1_AR in the presence or absence of 250 nM 8-CPT and 250 nM FSCPX (**a**), and of ZM-241,385 on hA_2A_AR in the presence or absence of 90 nM MSX-2 and 15 nM LUF6632 (**b**). Graphs show mean values of one representative MS binding competition association experiment performed in duplicate

**Fig. 7:**
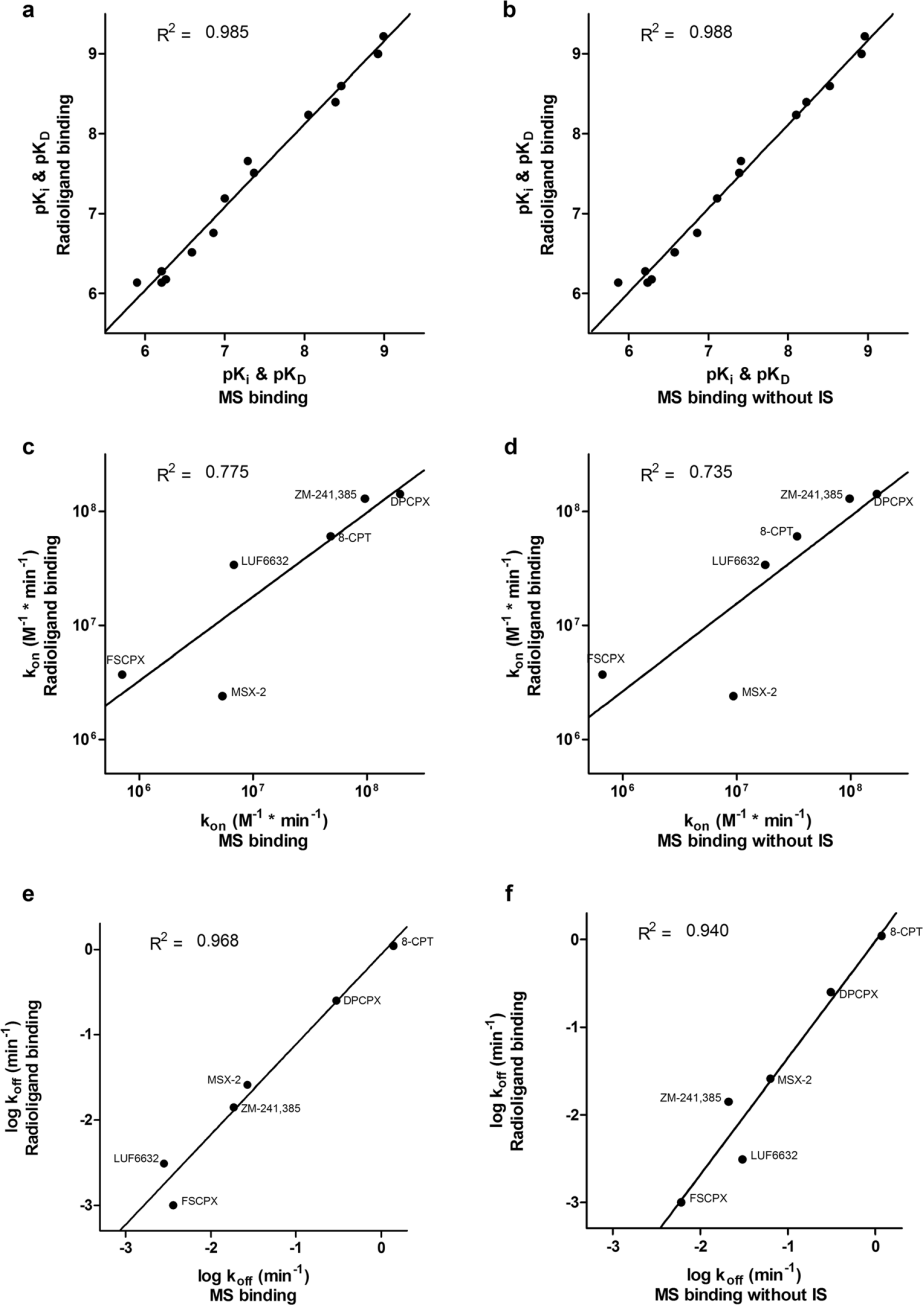
Correlation plots of results obtained by MS binding and radioligand binding assays on hA_1_AR and hA_2A_AR. Values of marker ligands DPCPX and ZM-241,385 were measured directly on their respective binding targets hA_1_AR and hA_2A_AR by saturation (**a**, **b**), association (**c**, **d**), and dissociation (**e**, **f**) assays, while values of the competing ligands were measured indirectly by displacement (**a**, **b**) and competition association assays (**c**–**f**). Affinity values in p*K*_D_ and p*K*_i_ (**a**, **b**), association rates in *k*_on_ (**c**, **d**), and dissociation rates in log *k*_off_ (**e**, **f**) were compared. Correlation plots **a**, **c**, and **e** show MS binding results standardized with deuterium-labeled internal standard, while **b**, **d**, and **f** show truly label-free MS binding results without internal standard. Data points represent mean values of at least three separate experiments performed in duplicate. *R* ^2^ values were calculated by linear regression performed on log-transformed values

DPCPX and ZM-241,385 were used as marker ligands for the MS binding assays. These marker ligands are also available as well-established tritium-labeled radioligands with high affinity for respectively the hA_1_AR and hA_2A_AR, which made it possible to validate the MS binding assays. For the displacement assays, the competing ligands on the hA_1_AR were CPA (selective agonist), 8-CPT (selective antagonist), ZM-241,385 (hA_2A_AR-selective antagonist), and NECA (non-selective agonist), and on the hA_2A_AR, they were UK-432,097 (selective agonist), MSX-2 (selective antagonist), DPCPX (hA_1_AR-selective antagonist), and NECA. For the competition association assays, the competing ligands on the hA_1_AR were 8-CPT (fast dissociation) and FSCPX (irreversibly binding to hA_1_AR resulting in an apparent slow dissociation), and on the hA_2A_AR, they were MSX-2 (fast dissociation) and LUF6632 (slow dissociation). Marker ligand concentrations that were found in the eluates of the MS binding assays ranged from 31 to 242 pM for DPCPX and 42 to 289 pM for ZM-241,385 (Fig. 2). The MS binding data in Figs. 3, 4, 5, and 6 and Tables 2, 3, 4, 5, and 6 is based on data with deuterium-labeled internal standard compensation of the marker ligand peak area (M/IS), except when stated otherwise in Tables 5 and 6. In Fig. 7, both M/IS-based and marker peak area-based (without internal standard compensation and thus completely unlabeled) data are compared with radioligand binding data.

**Table 2:** Affinity and *B*_max_ values of DPCPX for the hA_1_AR and ZM-241,385 for the hA_2A_AR as determined in MS binding and radioligand binding saturation assays. Values are mean *K*_D_ in nM ± SEM and mean *B*_max_ in pmol/mg protein ± SEM of at least three independent experiments performed in duplicate

	*K*_D_ ± SEM (nM)		*B*_max_ (pmol/mg protein)	
	MS binding	Radioligand binding	MS binding	Radioligand binding
DPCPX on hA_1_AR	3.43 ± 0.02	2.5 ± 0.1 [29]	17.3 ± 0.3	14 ± 1 [29]
ZM-241,385 on hA_2A_AR	1.03 ± 0.07	0.60 ± 0.07 [23]	2.3 ± 0.3	1.9 ± 0.4 [23]

**Table 3:** Affinity (*K*_i_ values) of CPA, 8-CPT, ZM-241,385, and NECA as determined in MS binding and radioligand binding displacement assays on hA_1_AR. The displacement curves of CPA and NECA fitted to a two-state site binding model, which yielded high and low binding affinities for the receptor. Values are mean *K*_i_ in nM ± SEM of at least three independent experiments performed in duplicate

	*K*_i_ ± SEM (nM)	
	MS binding	Radioligand binding
CPA	139 ± 32	175 ± 13
CPA-high	1.2 ± 0.4	1.0 ± 0.5
CPA-low	256 ± 81	304 ± 23
8-CPT	43 ± 11	31 ± 1
ZM-241,385	619 ± 78	523 ± 13
NECA	616 ± 76	731 ± 94 [22]
NECA-high	4.1 ± 1.4	4 ± 1 [22]
NECA-low	1273 ± 56	731 ± 94 [22]

**Table 4:** Affinity (*K*_i_ values) of UK-432,097, MSX-2, DPCPX, and NECA as determined in MS binding and radioligand binding displacement assays on hA_2A_AR. Values are mean *K*_i_ in nM ± SEM of at least three independent experiments performed in duplicate

	*K*_i_ ± SEM (nM)	
	MS binding	Radioligand binding
UK-432,097	52 ± 1	22 ± 5 [23]
MSX-2	9.0 ± 0.3	5.7 ± 0.1
DPCPX	550 ± 141	667 ± 77
NECA	100 ± 12	64 ± 1 [23]

**Table 5:** Association and dissociation rates of DPCPX, 8-CPT, and FSCPX determined in MS binding and radioligand binding assays on the hA_1_AR. MS binding (M/IS) values were obtained by analysis with compensation by internal standard, just as the MS binding values in Tables 2, 3, and 4. MS binding (marker) values were obtained by analysis of marker chromatograms solely, without compensation by internal standard, and thus label-free. The kinetic values of DPCPX were determined by association and dissociation assays, while the kinetic values of 8-CPT and FSCPX were determined by competition association assays with 6 nM DPCPX as marker ligand. Values are mean *k*_on_ in M^−1^ min^−1^ ± SEM and mean *k*_off_ in min^−1^ of at least three independent experiments performed in duplicate

	*k*_on_ ± SEM (M^−1^ min^−1^)			*k*_off_ ± SEM (min^−1^)		
	MS binding (M/IS)	Radioligand binding	MS binding (marker)	MS binding (M/IS)	Radioligand binding	MS binding (marker)
DPCPX	2.0 ± 0.3 × 10^8^	1.4 ± 0.2 × 10^8^ [29]	1.7 ± 0.4 × 10^8^	0.29 ± 0.02	0.25 ± 0.01 [29]	0.31 ± 0.02
8-CPT	5 ± 2 × 10^7^	6 ± 2 × 10^7^	3 ± 2 × 10^7^	1.4 ± 0.5	1.1 ± 0.3	1.2 ± 0.8
FSCPX^a^	0.7 ± 0.2 × 10^6^	3.7 ± 1.0 × 10^6^ [29]	0.7 ± 0.2 × 10^6^	0.004 ± 0.002	0.0010 ± 0.0002 [29]	0.006 ± 0.004

^a^Apparent kinetic values were calculated for covalently binding FSCPX

**Table 6:** Association and dissociation rates of ZM-241,385, MSX-2, and LUF6632 determined in MS binding and radioligand binding assays on the hA_2A_AR. MS binding (M/IS) values were obtained by analysis with compensation by internal standard, just as the MS binding values in Tables 2, 3, and 4. MS binding (marker) values were obtained by analysis of marker chromatograms solely, without compensation by internal standard, and thus label-free. The kinetic values of ZM-241,385 were determined by association and dissociation assays, while the kinetic values of MSX-2 and LUF6632 were determined by competition association assays with 3 nM ZM-241,385 as marker ligand. Values are mean *k*_on_ in M^−1^ min^−1^ ± SEM and mean *k*_off_ in min^−1^ of at least three independent experiments performed in duplicate

	*k*_on_ ± SEM (M^−1^ min^-1^)			*k*_off_ ± SEM (min^−1^)		
	MS binding (M/IS)	Radioligand binding	MS binding (marker)	MS binding (M/IS)	Radioligand binding	MS binding (marker)
ZM-241,385	9.5 ± 0.7 × 10^7^	13 ± 6 × 10^7^ [14]	9.8 ± 2.0 × 10^7^	0.019 ± 0.002	0.014 ± 0.003 [14]	0.021 ± 0.005
MSX-2	5.4 ± 0.6 × 10^6^	2.4 ± 0.2 × 10^6^	9.3 ± 1.3 × 10^6^	0.027 ± 0.005	0.026 ± 0.004	0.063 ± 0.022
LUF6632	0.7 ± 0.1 × 10^7^	3.4 ± 0.4 × 10^7^ [14]	1.8 ± 0.7 × 10^7^	0.0028 ± 0.0001	0.0031 ± 0.0002 [14]	0.030 ± 0.011

For the validation of MS binding assays, radioligand binding data that was published previously by our group was used. In the case that no in-house radioligand binding data was available, the concerning assays were performed following previously established protocols [22, 23]. Radioligand binding data for the marker ligands DPCPX and ZM-241,385 on their respective targets from saturation, association, and dissociation assays was published previously (Table 2). Displacement and competition association radioligand binding data of the competing ligands NECA (displacement on hA_1_AR and hA_2A_AR), UK-432,097 (displacement on hA_2A_AR), FSCPX (competition association on hA_1_AR), and LUF6632 (competition association on hA_2A_AR) was available as well from previous publications (Tables 3, 4, 5, and 6). Newly acquired radioligand binding data was from radioligand displacement assays with CPA, 8-CPT, and ZM-241,385 on the hA_1_AR; radioligand displacement assays with MSX-2 and DPCPX on the hA_2A_AR; and radioligand competition association assays with 8-CPT on the hA_1_AR and with MSX-2 on the hA_2A_AR.

MS binding saturation of the marker ligands DPCPX and ZM-241,385 to the hA_1_AR and hA_2A_AR, respectively, fitted a one-site saturation binding model (Fig. 3). DPCPX had an affinity of 3.43 ± 0.02 nM and a *B*_max_ of 17.3 ± 0.3 pmol/mg protein for the hA_1_AR which was well in accordance with the previously found data from radioligand binding assays of 2.5 ± 0.1 nM and 14 ± 1 pmol/mg protein, respectively (Table 2). The same was true for ZM-241,385 with an MS binding affinity of 1.03 ± 0.07 nM and *B*_max_ of 2.3 ± 0.3 pmol/mg protein for the hA_2A_AR, compared to a radioligand binding affinity of 0.60 ± 0.07 nM and *B*_max_ of 1.9 ± 0.4 pmol/mg protein. The displacement of marker ligands DPCPX and ZM-241,385 binding from the hA_1_AR and hA_2A_AR by their competing ligands fitted well to either one-state or two-state ligand binding displacement models (Fig. 4). The affinities found in MS binding displacement assays for the competing ligands CPA, 8-CPT, ZM-241,385, and NECA for the hA_1_AR (Table 3) and UK-432,097, MSX-2, DPCPX, and NECA for the hA_2A_AR (Table 4) were in good agreement to the radioligand binding assays. The two-state binding model fits observed for the agonists CPA and NECA on the hA_1_AR were observed in radioligand binding assays as well, and the resulting high and low affinity values were in good agreement (Table 3). The association and dissociation of marker ligands DPCPX and ZM-241,385 to the hA_1_AR and hA_2A_AR fitted well to one-phase association and dissociation models (Fig. 5), and the resulting association and dissociation rates were in good agreement between MS binding and radioligand binding assays (Tables 5 and 6).

With the association and dissociation rates validated for the marker ligands, MS binding competition association assays were performed. The competition association curves in the presence of FSCPX (hA_1_AR) and LUF6632 (hA_2A_AR) yielded an “overshoot” shape typical for slowly dissociating ligands, while in the presence of 8-CPT (hA_1_AR) and MSX-2 (hA_2A_AR), the curves were typical for fast-dissociating ligands (Fig. 6). FSCPX displaced the marker ligand DPCPX completely after 120 min. The association rates of 8-CPT and MSX-2 agreed well between MS binding and radioligand binding assays, but less so in case of FSCPX and LUF6632 (Tables 5 and 6). The dissociation rates of 8-CPT, MSX-2, and LUF6632 were in good agreement as well, but not the apparent dissociation rate of FSCPX.

Linear regression performed on the correlation plots of MS binding data (based on M/IS detection) against radioligand binding data yielded the following coefficients of determination and equations: *R*^2^ = 0.985 and *y* = 1.04*x* − 0.199 (*K*_i_ and *K*_D_, Fig. 7a), *R*^2^ = 0.775 and *y* = 0.738*x* + 2.09 (*k*_on_, Fig. 7c), and *R*^2^ = 0.968 and *y* = 1.06*x* − 0.0550 (*k*_off_, Fig. 7e). Similar correlation plots of MS binding data without IS compensation, solely based on marker peak area, against radioligand binding data yielded *R*^2^ = 0.988 and *y* = 1.05*x* − 0.299 (*K*_i_ and *K*_D_, Fig. 7b), *R*^2^ = 0.735 and *y* = 0.767*x* + 1.82 (*k*_on_, Fig. 7d), and *R*^2^ = 0.940 and *y* = 1.33*x* − 0.0168 (*k*_off_, Fig. 7f).

## Discussion

### Preparation of internal standards

Including an internal or external standard is a good practice in mass spectrometry, to compensate for ion suppression by matrix effects from cell contents, sample evaporation, and instrumental drift [10]. We used the internal standard method as this is the most accurate manner to compensate for these sources of signal distortion and to increase the accuracy of MS methods. Preferably, the internal standard is a molecule with the same chemical properties as the molecule of interest but with a distinct mass. Hence, deuterated DPCPX (d4) and ZM-241,385 (d4) were synthesized to serve as internal standard for the MS binding assays on hA_1_AR and hA_2A_AR, respectively. The resulting mass difference between the parent compounds and their internal standards ensured minimal signal overlap by their isotope patterns. For the synthesis of [^2^H_4_]ZM-241,385 5, the pure isotopologue [^2^H_4_]tyrosine (SI 14) was commercially available as precursor for the [^2^H_4_]tyramine 4 building block. For the synthesis of [^2^H_4_]DPCPX 2, the building block 1 was deuterated in-house by a rhodium-catalyzed reduction of two allylic double bonds in the presence of deuterium gas generated in situ. During this process, deuterium-hydrogen scrambling occurred which resulted in a mixture of isotopologues [^2^H_0_]DPCPX to [^2^H_8_]DPCPX in a Gaussian distribution as a final product. This had no negative influence on the results of the MS Binding assays, since the masses of parent ions and fragments of the most abundant isotopologue [^2^H_4_]DPCPX 5 were selected for quantification.

### MS binding assays

Affinity, association, and dissociation rates measured directly for the marker ligands DPCPX on the hA_1_AR and ZM-241,385 on the hA_2A_AR were in good agreement to the values found in radioligand binding assays (Figs. 3 and 5, Tables 2, 5, and 6). The good performance of the MS binding saturation, association, and dissociation assays in which solely the marker ligand and no competing ligand was present was a prerequisite to continue with the MS binding displacement and competition association assays.

To demonstrate the MS binding displacement assays, a combination of selective and non-selective agonists and antagonists were chosen as competing ligands. For the hA_1_AR, these ligands were CPA, 8-CPT, ZM-241,385, and NECA, and for the hA_2A_AR, they were UK-432,097, MSX-2, DPCPX, and NECA. The determined affinity values were in good agreement between MS binding and radioligand binding assays for all these competing ligands (Fig. 4, Tables 3 and 4). Furthermore, the binding of agonists CPA and NECA to the hA_1_AR fitted to a pronounced two-phase displacement curve as was found before in radioligand binding assays.

Kinetic properties of ligands are of emerging interest and are thought to be important predictors of clinical performance [3, 26]. Therefore, we developed and validated MS binding competition association assays, by which kinetic properties of competing ligands can be analyzed by measuring the amount of bound marker ligand at different time points, in the presence of one concentration of these competing ligands. A fast and a slowly dissociating competing ligand were chosen for each target. Fast- and slow-dissociating ligands yield distinct characteristic competition association graphs, without and with overshoot, respectively. For the hA_1_AR, the ligands 8-CPT and FSCPX and for hA_2_AR, MSX-2 and LUF6632 were tested. 8-CPT and MSX-2 dissociate fast from their targets. FSCPX is an irreversibly binding antagonist selective for the hA_1_R [13, 27, 28] and thus yields the characteristic overshoot graph for slowly dissociating ligands [29], with the exception that it eventually displaces the marker ligand DPCPX completely (Fig. 6a). LUF6632 was characterized earlier as a slowly dissociating ligand selective for the hA_2A_AR [14].

Dissociation rates were in good agreement between the MS binding and radioligand binding competition association assays (Fig. 7e, Tables 5 and 6), with the exception of the apparent dissociation rate of the irreversibly binding FSCPX (Table 5). Association rates found for the competing ligands in competition association assays varied more, especially for the slowly or not at all dissociating ligands FSCPX and LUF6632 (Fig. 7c, Tables 5 and 6). It has to be noted that as it binds irreversibly to the hA_1_AR, FSCPX does not actually dissociate from the target. However, fitting the FSCPX data into the competition association model still enables the calculation of apparent association and dissociation rates. Being apparent values, they may vary between studies which could be an explanation for the diverging kinetic rates of FSCPX found in MS binding and radioligand binding assays (Table 5).

Altogether, these results validate the use of MS binding assays to determine affinity values and dissociation rates by saturation, association, dissociation, and competition association assays. However, association rate determination was only accurate by direct measurement on the marker ligands.

### Necessity of deuterium-labeled internal standard

As mentioned above, including an internal or external standard is a good practice in mass spectrometry. We used the internal standard method as this is the most accurate manner to compensate for sources of signal distortion. However, the use of a deuterium-labeled internal standard makes the MS binding assay a labeled assay, even if the marker ligand that binds to the target is itself unlabeled. For fast screening of new marker ligands, the use of an external standard or even no standard at all would be vastly advantageous, as the whole assay becomes an unlabeled assay. Moreover, to directly determine association and dissociation rates of non-labeled ligands would be an improvement over the use of competition association assays. Therefore, we compared the performance of the MS binding assay with and without compensation by deuterium-labeled internal standard. Although in the latter case the resulting graphs of each separate experiment were somewhat less accurate, *K*_i_ and *K*_D_ values could still be determined without loss of accuracy (Fig. 7b). The *k*_off_ values indirectly determined by the competition association assay correlated less well with radioligand binding assays, although retaining a good coefficient of determination (Fig. 7f). The determination of *k*_on_ values correlated less well with radioligand binding assays irrespective of the use of an internal standard (Fig. 7c, d). In contrast to this, the directly measured association and dissociation rates of marker ligands DPCPX and ZM-241,385 were still in good agreement with radioligand binding experiments (Tables 5 and 6).

## Conclusion

We developed and validated MS binding assays for the adenosine A_1_ and A_2A_ receptors. The results from ligand saturation, association, dissociation, and displacement assays were in good agreement with radioligand binding data. The results from competition association assays were in good agreement with radioligand binding data for dissociation rates but less so for association rates. Furthermore, we investigated the necessity to include deuterium-labeled internal standards in MS binding assays. Saturation, association, dissociation, and displacement assay results were still in good agreement with radioligand binding assays when the internal standard was not included. In competition association assays, the inclusion of an internal standard was beneficial for good correlation of dissociation rates with radioligand binding data. However, by excluding the use of internal standards in MS binding assays, it would be relatively simple to measure association and dissociation rates of a number of unlabeled ligands directly, without the need for competition association assays. We conclude that the use of deuterium-labeled internal standards is in this case unnecessary which makes the MS binding assay a truly unlabeled ligand binding assay. As this internal standard-free approach may be applied to other targets than the currently investigated adenosine A_1_ and A_2A_ receptors, we foresee the promising future application of MS binding to directly measure binding properties by saturation, association, and dissociation assays, without the use of any labeled internal standards.

## Electronic supplementary material

ESM 1 (PDF 422 kb).
