# Supplementary material for: Mass spectrometry-based ligand binding assays on adenosine A1 and A2A receptors
Source: Purinergic Signal. 2015 Oct 19;11(4):581–94. doi: 10.1007/s11302-015-9477-0 (PMC4648803; doi:10.1007/s11302-015-9477-0)
Supplement: Supplementary file 1 — (PDF 422 kb). [file 11302_2015_9477_MOESM1_ESM.pdf]

Supplementary Information

Mass spectrometry-based ligand binding assays on adenosine A_1 and A_{2A} receptors

Purinergic Signalling

A. Massink, M. Holzheimer, A. Hölscher, J. Louvel, D. Guo, G. Spijksma, T. Hankemeier, A. P. IJzerman

Division of Medicinal Chemistry, LACDR, Leiden University, Leiden, The Netherlands (A.M., M.H., A.H., J.L., D.G., A.P.IJ.); Division of Analytical Biosciences, LACDR, Leiden University, Leiden, The Netherlands (G. S., T.H.)

Corresponding author: Prof. Dr. Adriaan P. IJzerman, ijzerman@lacdr.leidenuniv.nl

Synthesis of deuterated internal standards [^{2}H]DPCPX and [^{2}H]ZM-241,385

Results

*Synthesis of [$^2H_4$]DPCPX **2***

[$^2H_4$]DPCPX **2** was prepared according to the synthetic route shown in Scheme 3 and was adopted from previously described syntheses of non-deuterated DPCPX. Starting from the commercially available diallyl urea **6**, 1,3-diallyl-6-aminouracil **7** was synthesized by condensation with cyanoacetic acid and subsequent base promoted ring closure [1]. **7** was then nitrosylated on the 5-position and subsequently reduced to the diamine **8** [2]. Cyclization of **8** with cyclopentanecarbonyl chloride gave the [$^2H_4$]DPCPX precursor **1** [3]. Finally, the allylic double bonds of compound **1** were reductively deuterated in the presence of Wilkinson's catalyst with $NaBD_4$ as a deuterium source generating deuterium gas *in situ* upon addition of D_2O [7]. The mass spectrum showed a mass range for the ($M+H^+$) species from 305.20 ([$^2H_0$] isotopologue) to 313.27 ([$^2H_8$] isotopologue) in a Gaussian distribution with the desired [$^2H_4$]DPCPX **2** as most abundant isotopologue generating the main mass peak at 309.33**.**

*Synthesis of [$^2H_4$]ZM-241,385 **5***

[$^2H_4$]ZM-241,385 **5** was prepared according to the synthetic route shown in Scheme 4 and was adopted from previously described syntheses of non-deuterated ZM-241,385. Commercially available ethyl furan-2-carboxylate **9** was reacted with hydrazine hydrate to give furan-2-carbohydrazide **10.** Through a substitution reaction of **10** with methyl isothiourea hemisulfate diamide **11** was obtained, which underwent a cyclization in water to give the 1,2,4-triazole **12**. In a subsequent cyclization of **12** with dimethyl cyanocarbonimidothioate the triazolotriazine **13** was obtained [4] and the corresponding methylsulfone **3** was prepared by oxidation of the methylthioether with *m*CPBA [5]. For the synthesis of the deuterated building block, [$^2H_4$]tyrosine **14** was enzymatically decarboxylated using tyrosine decarboxylase and pyridoxal-5-phosphate as cofactor to give [$^2H_4$]tyramine **4** [6]. Finally, reaction of the deuterated tyramine **4** with methylsulfone compound **3** yielded the final product [$^2H_4$]ZM-241,385 **5** [4]. MS analysis showed a mass of 342.7 ($M+H^+$) and confirmed the incorporation of 4 deuterium atoms in the final product.

Schemes

Scheme 3 Synthesis of [$^2H_4$]DPCPX (**2**). Reagents and conditions: *i* 1. Cyanoacetic acid, Ac_2O, MW, 80 °C, 4 h, 2. EtOH, NaOH 2M; *ii* 1. $NaNO_2$, 42% AcOH, rt, 1 h, 2. $Na_2S_2O_4$, EtOAc/H_2O = 2/1, rt, 0.5 h, *iii* 1. Cyclopentanecarbonyl chloride, pyridine, dry CH_2Cl_2, N_2-atm, 5 °C to rt, 2 h, 2. NaOH 3M, reflux, 1 h; *iv* $Rh(PPh_3)_3Cl$, $NaBD_4$, D_2O, dry THF, 60 °C, 3.5 h

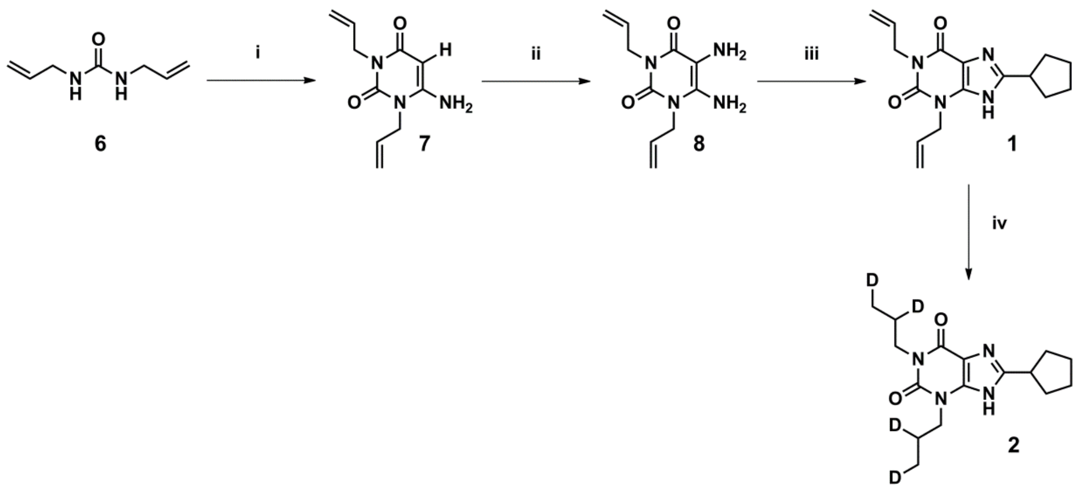

Scheme 4 Synthesis of [$^2H_4$]ZM-241,385 (**5**). Reagents and conditions: *i* Hydrazine hydrate, EtOH, reflux, 18 h; *ii* Methyl isothiourea hemisulfate, 1% NaOH, rt, 23 h; *iii* H_2O, MW, 160 °C, 1.5 h; *iv* Dimethyl cyanocarbonimidodithioate, 180 °C, 16 h; *v m*CPBA, CH_2Cl_2, –5 °C to rt, 18 h; *vi* Tyr-decarboxylase, pyridoxal-5-phosphate, acetate buffer 0.1 M (pH 5.5), H_2O, 37 °C, 17 h; *vii* Et_3N, MeCN, MW, 70 °C, 3 h

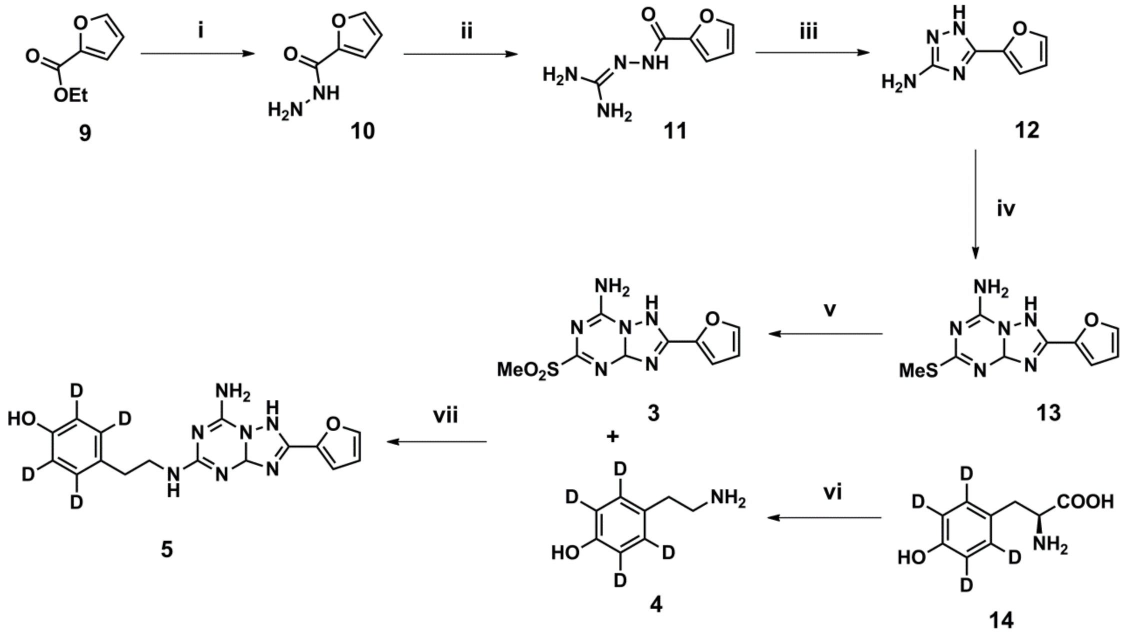

References

1. Devi I, Bhuyan PJ (2005) An expedient method for the synthesis of 6-substituted uracils under microwave irradiation in a solvent-free medium. Tetrahedron Lett 46 (34):5727-5729. doi:doi:10.1016/j.tetlet.2005.06.075
2. Soriano A, Ventura R, Molero A, Hoen R, Casadó V, Cortés A, Fanelli F, Albericio F, Lluis C, Franco R, Royo M (2009) Adenosine A_{2A} receptor-antagonist/dopamine D_2 receptor-agonist bivalent ligands as pharmacological tools to detect A_{2A}-D_2 receptor heteromers. J Med Chem 52 (18):5590-5602. doi:10.1021/jm900298c
3. Erickson RH, Hiner RN, Feeney SW, Blake PR, Rzeszotarski WJ, Hicks RP, Costello DG, Abreu ME (1991) 1,3,8-trisubstituted xanthines. Effects of substitution pattern upon adenosine receptor A_1/A_2 affinity. J Med Chem 34 (4):1431-1435
4. Jörg M, Agostino M, Yuriev E, Mak F, Miller N, White J, Scammells P, Capuano B (2013) Synthesis, molecular structure, NMR spectroscopic and computational analysis of a selective adenosine A_{2A} antagonist, ZM 241385. Struct Chem 24 (4):1241-1251. doi:10.1007/s11224-012-0151-7
5. Jörg M, Shonberg J, Mak FS, Miller ND, Yuriev E, Scammells PJ, Capuano B (2013) Novel adenosine A_{2A} receptor ligands: a synthetic, functional and computational investigation of selected literature adenosine A_{2A} receptor antagonists for extending into extracellular space. Bioorg Med Chem Lett 23 (11):3427-3433. doi:10.1016/j.bmcl.2013.03.070
6. Ntai I, Phelan VV, Bachmann BO (2006) Phosphonopeptide K-26 biosynthetic intermediates in Astrosporangium hypotensionis. Chem Commun (Camb) (43):4518-4520
7. Adair GRA, Kapoor KK, Scolan ALB, Williams JMJ (2006) Ruthenium catalysed reduction of alkenes using sodium borohydride. Tetrahedron Lett 47 (50):8943-8944. doi:10.1016/j.tetlet.2006.10.026
